# Practical implementation of true on-site water recycling systems for hand washing and toilet flushing

**DOI:** 10.1016/j.wroa.2020.100051

**Published:** 2020-04-08

**Authors:** Eva Reynaert, Esther E. Greenwood, Bonginkosi Ndwandwe, Michel E. Riechmann, Rebecca C. Sindall, Kai M. Udert, Eberhard Morgenroth

**Affiliations:** a Eawag, Swiss Federal Institute of Aquatic Science and Technology, 8600, Dübendorf, Switzerland; b ETH Zürich, Institute of Environmental Engineering, 8093, Zürich, Switzerland; c University of KwaZulu Natal, Pollution Research Group, 4041, Durban, South Africa

**Keywords:** Field test, Water recycling, Hand washing, Toilet flushing, Wastewater reuse, Biologically activated membrane bioreactor (BAMBi)

## Abstract

On-site wastewater reuse can improve global access to clean water, sanitation and hygiene. We developed a treatment system (aerated bioreactor, ultrafiltration membrane, granular activated carbon and electrolysis for chlorine disinfection) that recycles hand washing and toilet flush water.

Three prototypes were field-tested in non-sewered areas, one in Switzerland (hand washing) and two in South Africa (hand washing, toilet flushing), over periods of 63, 74 and 94 days, respectively.

We demonstrated that the system is able to recycle sufficient quantities of safe and appealing hand washing and toilet flush water for domestic or public use in real-life applications. Chemical contaminants were effectively removed from the used water in all prototypes. Removal efficiencies were 99.7% for the chemical oxygen demand (COD), 98.5% for total nitrogen (TN) and 99.9% for phosphate in a prototype treating hand washing water, and 99.8% for COD, 95.7% for TN and 89.6% for phosphate in a prototype treating toilet flush water. While this system allowed for true recycling for the same application, most on-site wastewater reuse systems downcycle the treated water, i.e., reuse it for an application requiring lower water quality. An analysis of 18 selected wastewater reuse specifications revealed that at best these guidelines are only partially applicable to innovative recycling systems as they are focused on the downcycling of water to the environment (e.g., use for irrigation). We believe that a paradigm shift is necessary and advocate for the implementation of risk-based (and thus end-use dependent) system performance targets to evaluate water treatment systems, which recycle and not only downcycle water.

## Highlights

• We developed an on-site system that recycles hand washing and toilet flush water.
• Three prototypes were field-tested in Switzerland and South Africa.
• The prototypes produced sufficient, safe and appealing recycled water.
• We removed 99.8% of the oxygen demand and 95.7% of the nitrogen from flush water.
• True water recycling was demonstrated in real-life applications.

## Graphical abstract

**Figure:**
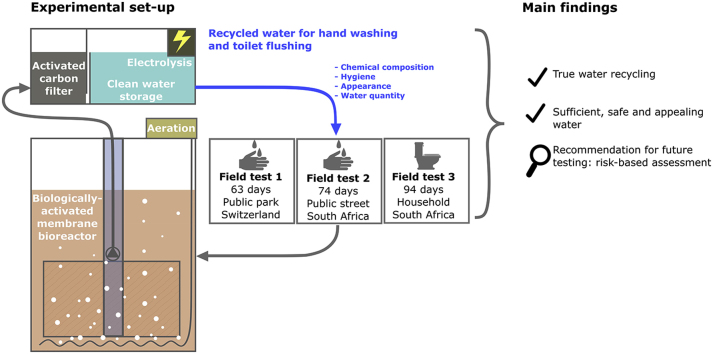

## Abbreviations and terminology

BAMBi: Biologically Activated Membrane Bioreactor
COD: Chemical Oxygen Demand
CWT: Clean Water Tank
DOC: Dissolved Organic Carbon
Downcycling: Water reuse for an application requiring lower water quality
GDM: Gravity-Driven Membrane
HRT: Hydraulic Retention Time
Recycling: Water reuse for the same application
TN: Total Nitrogen
UD(D)T: Urine-Diverting (Dry) Toilet
WASH: Water, Sanitation and Hygiene
WWT: Wastewater Tank

## 1 Introduction

The United Nations has reported that nearly 850,000 people die every year from lack of access to clean water, sanitation and hygiene (WASH, WHO and UNICEF, 2017). WASH challenges are interdependent: access to safe water, improved sanitation facilities or washing facilities do not, independently of one another, necessarily lead to improved health (Eisenberg et al., 2007). Consequently, adequate sanitation facilities should not only protect water sources from contamination, but also provide access to improved hygiene. In particular, hand washing with water and soap has been shown to be at least as important as drinking water quality in reducing the burden of global infectious diseases (Wagstaff et al., 2006). Reliable water supplies are also required for flush-based sanitation systems, which are, to this day, still what most people aspire to (Flores, 2011). One of the great challenges for the provision of WASH services is to provide, process, and distribute the required water.

Today, two-thirds of the global population experience severe water scarcity at least one month per year (Mekonnen and Hoekstra, 2016). Additionally, aging infrastructure, population growth, rapid urbanization and low institutional reliability acutely challenge the practicability of centralized water treatment, distribution and evacuation networks in many parts of the world (Larsen et al., 2016). Many authorities responsible for providing WASH services face the challenge of insufficient freshwater supplies and infrastructure with which to meet their citizens’ increasing expectations. Innovative approaches are necessary to provide locally adapted, resource-efficient solutions.

The Blue Diversion Autarky project (www.autarky.ch) aims to fill this gap by developing decentralized sanitation systems that are safe, affordable and aspirational. As part of this project, we devised a true on-site water recycling system called the Water Wall. The system treats and recycles water for applications like hand washing and toilet flushing without the need for external water input. The combination of an aerobic bioreactor with ultrafiltration membrane, activated carbon filter and electrolysis flow cell provides multiple barriers for chemical and microbial contaminants. Previously published laboratory studies have demonstrated that the system is capable of removing bacteria (Nguyen et al., 2017; Ziemba et al., 2019), nutrients (Ziemba et al., 2018) and color (Künzle et al., 2015) from recycled water.

A range of treatment technologies has been suggested to reuse toilet flush water on-site, where reuse can imply water recycling (water reuse for the same application) or water downcycling (water reuse for a lower-quality application). Some systems use electrochemical processes to treat the water (e.g., Cid et al., 2018; Rogers et al., 2018; Sahondo et al., 2020), but most systems rely on anaerobic (e.g., Bair et al., 2015) or aerobic membrane bioreactors (e.g., Abegglen et al., 2008; Fountoulakis et al., 2016; Ratanatamskul et al., 2014; Santasmasas et al., 2013). Many of these systems were capable of partially or completely meeting treatment targets from selected wastewater reuse specifications in real-life testing where the water was downcycled. But the treated water quality in these systems would not be sufficient for long-term true recycling for the same application. There is a diversity of regional and national guidelines with a diversity of quality targets for reuse. Recently, risk-based frameworks for water reuse have been introduced in the ISO 30500 on non-sewered sanitation systems and the WERF framework for the development of health guidance for decentral water reuse (ISO, 2018; Sharvelle et al., 2017).

In this study, we present field-testing results for three Water Wall prototypes that were implemented in selected locations in Switzerland and South Africa, where they provided recycled hand washing and flush water to diverse focus groups. The purpose was to show that the Water Wall system is capable of providing sufficient water that is suitable for the intended use, even under highly variable and unpredictable real-life usage conditions. Our approach was to verify that (i) the chemical composition allows for its repeated use for toilet flushing and hand washing: low and stable oxygen demand, nitrogen and phosphorus concentrations in the treated water, (ii) the microbial risk is low: no pathogen indicators detected and free residual chlorine in the storage tank, and (iii) the appearance is appealing: no color, turbidity or suspended solids visible to the human eye.

## 2 Methodology

### 2.1 Water Wall system

**Fig. 1:**
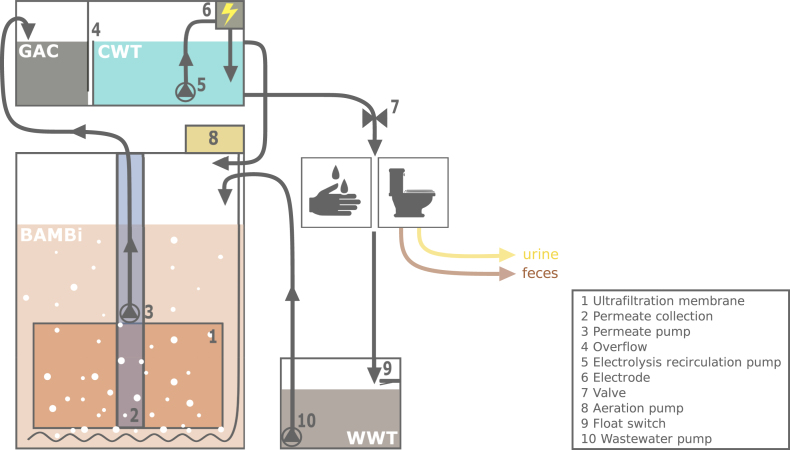
Process diagram of the Water Wall system, with the biologically activated membrane bioreactor (BAMBi) on the bottom and the granular activated carbon (GAC) filter and clean water tank (CWT) on top. Water from the CWT is used for toilet flushing or hand washing, or recirculated to the BAMBi through an overflow. Depending on the specific setup, a wastewater tank (WWT) is necessary for the collection of the used water.

The Water Wall systems (Fig. 1) consisted of two main components: the core treatment took place in a biologically activated membrane bioreactor (BAMBi, Künzle et al., 2015), after which the water was polished and disinfected in a clean water tank (CWT).

The BAMBi contained a standing sandwich membrane module (Microclear MCXL, Newterra, Langgöns, Germany) featuring a 150 kDa polyethersulfone ultrafiltration membrane (Microdyn-Nadir, Wiesbaden, Germany). Aeration was provided directly below the membrane module. At low aeration rates, incomplete mixing and formation of anoxic zones at the bottom of the reactor allow for simultaneous nitrification and denitrification (Ravndal et al., 2015). The BAMBi was operated in a gravity-driven membrane (GDM) configuration, in which the pressure on the membrane is supplied only by the water head (Peter-Varbanets et al., 2010). Active biomass in the BAMBi responsible for carbon oxidation, nitrification and denitrification is partially suspended and partially growing as a biofilm on the membranes. As discussed in Ravndal et al. (2015), long-term stable operation does not require systematic sludge wastage and does not require a specific solids retention time, greatly simplifying operation. During the three field tests described in this paper, sludge was never removed from the BAMBi. Water that passed through the membrane was collected in a permeate reservoir (10 cm polyvinyl chloride pipe connected to the membrane module permeate outlet, holding volume of 4 L), from where it was pumped through a granular activated carbon (GAC) filter (Norit 830, ∼1.5 mm grain diameter, Cabot, Boston, USA) to the CWT at regular intervals. In the CWT, water was recirculated at a rate of 0.5 L/min through an 85 cm^2^ boron-doped diamond electrolysis flow cell (DiaClean® 103, WaterDiam, Delémont, Switzerland) operated with polarity reversal. The electrolysis treatment produced residual chlorine from the chloride dissolved in the water. The target chloride concentration was 200 mg/L and chloride was provided through NaCl supplementation if necessary. From the CWT, the water was used for hand washing and toilet flushing, or recirculated back to the BAMBi through an overflow.

After use, the hand washing water was recirculated directly into the BAMBi by means of gravity, or collected in a wastewater tank (WWT), from where it was pumped into the BAMBi for treatment. The flush water provided from a urine-diverting toilet (UDT, save! Keramik Laufen AG, Laufen, Switzerland) passed through a solids separator (Separator, Aquatron, Västerås, Sweden) before collection in the WWT. The Water Wall was designed as part of a modular system and can be combined with different types of urine or feces treatment technologies.

Three Water Wall prototypes were tested in the field. The main characteristics are summarized in Table 1. Prototype 1 (P1) recycled up to 75 L/day of hand washing water, Prototype 2 (P2) recycled up to 500 L/day of hand washing water and Prototype 3 (P3) recycled up to 500 L/day of flush water (design capacities). P1 was initially filled with 65 L of tap water and inoculated with 2 L of activated sludge (from a conventional activated sludge wastewater treatment plant in Switzerland). P2 and P3 were initially filled with 360 L of tap water and inoculated with 10 L of activated sludge (from a conventional activated sludge wastewater treatment plant in South Africa). As the ratios of nutrients to carbon in hand washing water is generally too low for biological treatment, the users of P1 and P2 were provided with nutrient-supplemented biodegradable soap (Ziemba et al., 2019). The soap composition is detailed in the Supplementary Information Table S1.

**Table 1:** Overview of Water Wall prototypes. BAMBi: biologically activated membrane bioreactor. GAC: granular activated carbon filter. CWT: clean water tank. WWT: wastewater tank.

	Prototype 1 (P1) Hand Washing Station	Prototype 2 (P2) Hand Washing Station	Prototype 3 (P3) Toilet System
**Type of water recycled**	Hand washing water	Hand washing water	Flush water provided from a urine-diverting toilet with separation of the solids from the water
**Application**	Public	Public	Private
**Design capacity**			
Water production [L/day]	75	500	500 (10-people household)
**Tank volumes**			
BAMBi [L]	52	327	327
GAC [L]	6	15	15
CWT [L]	15	41	41
WWT [L]	None	20	20
**Aeration**			
Aeration rate [Nm^3^/h]	0.3	0.6	0.6
**Electrolysis operation**			
Voltage [V]	12	12	12
Current [mA]	100	1200	1200
Operation time t_ON_/t_OFF_ [s/s]	60/90, 60/60^a^	120/0, 60/120^b^	120/0, 120/60^b^
**Permeate pump operation**			
Operation time t_ON_/t_OFF_ [s/s]	10/300	10/250	15/300
Operation mode	Permeate pumped from bottom of membrane (membrane could dry out in case of water loss)	Permeate pumped from top of membrane (membrane cannot dry out in case of water loss)	Permeate pumped from top of membrane (membrane cannot dry out in case of water loss)
**Wastewater collection**	Wastewater flows from sink into BAMBi by means of gravity (no pump)	Wastewater is collected in 20 L tank and pumped into BAMBi when full (float switch)	Wastewater is collected in 20 L tank and pumped into BAMBi when full (float switch)
**Additives**	Nutrient-supplemented biodegradable soap	Nutrient-supplemented biodegradable soap	Biodegradable soap

a Change of settings to reach higher residual chlorine concentrations.

b Change of settings after replacement of electrolysis recirculation pump (increased flux to 3 L/min).

### 2.2 Testing contexts

The hand washing station P1 was tested in a public park in Zurich, Switzerland, next to an existing composting toilet and two urinals. The site was not connected to the power grid or to a sewer. Power was provided through a solar panel and a methanol fuel cell (EFOY Pro, Brunnthal, Switzerland). Testing took place over 63 days between May and July 2018.

The hand washing station P2 was tested in an informal settlement in Durban, South Africa, with approximately 500 households. The community relied on communal ablution blocks for access to water and sanitation. The hand washing station was located adjacent to the main road, with many people passing by the station on the way to or from town. Electric power was available on-site. Testing took place over 74 days between April and June 2019.

P3 was integrated in a toilet system, which was tested in a 14-person household in a peri-urban zone of Durban, South Africa. The prototype was installed next to an existing urine-diverting dry toilet (UDDT) that the household owned as part of a municipal cost-free basic sanitation program. The prototype was tested in a complete toilet system, with concurrent testing of the user interface and a urine treatment system developed as part of the Autarky project (more information on the process: www.autarky.ch). Feces were collected in a sealed container that was discharged off-site into a sewer twice per week. Testing took place over 94 days between March and June 2019.

Pictures of the three prototypes are shown in the Supplementary Information Fig. S2. All prototypes were removed from the sites after the end of the testing.

The present paper evaluates the functioning of the Water Wall system from a technical point of view. The field test was closely studied by a team of social scientists. Users’ interactions with the system and their acceptance of recycled water for hand washing and toilet flushing is outside the scope of this paper and will be discussed in separate publications.

### 2.3 Usage, water availability, water refill, flow rate through the membrane

P1 was equipped with a photoelectric sensor (EX-10, Panasonic, Kadoma, Japan) that allowed the number of usages to be logged. A two-day usage count was performed for data validation. The usage of P2 was recorded during a two-day usage count. P3 was equipped with miniature switches behind the flush buttons to count the number of toilet usages. These counts were validated through a 14-day self-reported survey by the users.

The water levels in the BAMBi and CWT were continuously monitored with pressure transducers (P1: PMP131, P2 and P3: PMC11, Endress + Hauser, Reinach, Switzerland). The volume of water to be refilled (due to losses from evaporation, losses at the tap, and losses at the solids/urine separation) was calculated based on the pressure readings. Water was refilled around 1x/week.

The flow rate through the membrane was calculated as the ratio between the total CWT volume and the time required to fill the CWT (empty to full).

### 2.4 Water quality monitoring

Samples from the CWT were taken bi-weekly and analyzed for their chemical composition, hygienic quality and appearance. For P2 and P3, weekly samples were taken from the WWT and analyzed for the chemical composition.

All samples were filtered at 0.45 μm (Nanocolor Chromafil membranefilter GF/PET 0.45 μm, Macherey-Nagel, Düren, Germany) for sample conservation and stored at 4 °C before chemical analysis. For P1, dissolved organic carbon (DOC) was measured using a total organic carbon analyzer (Shimadzu TOC-L, Kyoto, Japan). Ammonium was measured using a colorimetric assay (Hach, Loveland, USA). Nitrite, nitrate, chloride and phosphate were measured by means of ion chromatography (Metrohm 881, Herisau, Switzerland). The pH value was measured with a handheld meter (pH 330i, WTW, Germany). For P2 and P3, the chemical oxygen demand (COD), ammonium, nitrite, nitrate, total nitrogen (TN), chloride and phosphate were measured using colorimetric assays (Spectroquant, Merck, Darmstadt, Germany). The pH value was measured with a benchtop meter (Sension MM374, Hach, Loveland, USA). Details on the specific kits used for the spectrophotometric tests can be found in the Supplementary Information Table S3.

To assess the hygienic quality of the water, chlorine was measured on-site using a portable spectrophotometer (DR 1900, Hach, Loveland, USA) with corresponding test kits (DPD, 0–2 mg/L free chlorine, Hach, Loveland, USA). Samples for microbial indicators were cooled for transport and analyzed immediately in the laboratory. For P1, *E. coli* was measured according to the US EPA Method 1603 (US EPA, 2014). For P2 and P3, *E. coli* and total coliforms were enumerated with an enzyme activity test (Colilert-18/Quanti-Tray, IDEXX Laboratories, Westbrook, USA).

The appearance of the water was monitored in P2 and P3, for which turbidity was measured with a turbidimeter (2100Q, Hach, Loveland, USA), color with a color test kit (Model CO1, Hach, Loveland, USA), and total suspended solids (TSS) according to the APHA standard method (APHA, 2005).

### 2.5 Calculation of water recycling rate and organic/nutrient removal efficiencies

The water recycling rate rcl was defined as the ratio between the used water-returning to the system and the total amount of used water (including losses):

(1) $$rcl=\left(V_{tot\;usage}-V_{tot\;loss}\right)/V_{tot\;usage}$$

with $V_{tot\;usage}$: total volume of water used for hand washing or toilet flushing and $V_{tot\;loss}$: total volume of water lost from the system.

The removal efficiencies of COD, TN and phosphate from the water were calculated based on mass balances of the compounds in the system water volume (BAMBi and CWT). The total mass M of the compound i (COD, TN or phosphate) in the water corresponds to the product of the concentration $C_{i}$ (measured in the CWT) and the system water volume V. The change of mass of compound i over the field test duration is equal to the difference between the incoming mass $M_{in,i}$ (from the used water), the outflowing mass $M_{out,i}$ (from water losses) and the removal of the compound in the system $Rem_{i}$ (degradation and transformations):

(2) $$\Delta M_{i}=M_{in,i}-M_{out,i}-Rem_{i}$$

with $\Delta M_{i}=\left(C_{CWT,i,fin}-C_{CWT,i,ini}\right)\cdot V$, $M_{in,i}=C_{WWT,i}\cdot V_{tot\;in}$ and $M_{out,i}=C_{CWT,i}\cdot V_{tot\;loss}$, where $V_{tot\;in}$: total volume of water entering the system through the WWT, and $V_{tot\;loss}$: total volume of water lost from the system. $V_{tot\;in}$ was estimated from the total volume of water used for toilet flushing or hand washing.

The removal efficiency $rem_{i}$ for compound i is defined as:

(3) $$rem_{i}=\frac{Rem_{i}}{M_{in,i}}=\frac{M_{in,i}-M_{out,i}-\Delta M_{i}}{M_{in,i}}$$

Removal efficiencies were not calculated for P1 as the influent load was not known (P1 did not include a WWT and the used water entered directly into the BAMBi).

### 2.6 Energy requirements

For P1, the energy requirements were continuously logged by the fuel cell. P2 and P3 were connected to electric meters. Energy requirements were corrected for devices that were not part of the treatment system (e.g., monitoring devices).

### 2.7 Data analysis

Data analysis was conducted in R (R Development Core Team, 2017) and figures were produced using the package ggplot2 (Wickham, 2016). Chemical analyses often revealed concentrations below the detection limit of the method used. These censored data were analyzed with the package NADA (Lee, 2017). Average concentrations were computed using regression by maximum likelihood estimation for left-censored data, assuming a Gaussian distribution of the data.

## 3 Results

### 3.1 System performance

#### 3.1.1 Usage, water availability, refill and recycling rate

**Fig. 2:**
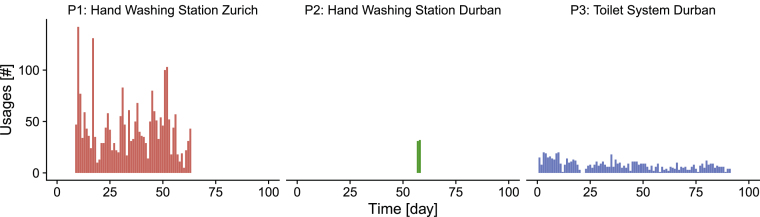
Daily usages. P1: online measurement with light barrier. P2: two-day counting campaign from 7:00 h to 19:00 h. P3: online measurement of flush button usage.

All systems were regularly used despite alternatives being available in the immediate surroundings. Fig. 2 presents the daily number of usages of each prototype. Average daily usages were 44 usages (22 L) for P1, 31.5 usages (31.5 L) for P2 and 8 usages (48 L) for P3. Especially in the case of P2 and P3, maximum usage was significantly below the design capacity (see Table 1), which may have an influence on the performance presented in the subsequent sections. We are nevertheless confident that P2 and P3 would have performed well at a usage closer to the capacity based on the experiences with P1.

**Fig. 3:**
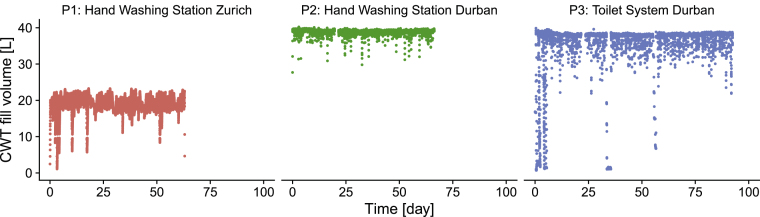
Fill volume in the clean water tank (CWT). P1: CWT volume 21 L; P2 and P3: CWT volume 41 L.

The CWT was usually full in all systems, as the tank was continuously refilled and unused water recirculated to the BAMBi (Fig. 3). Water was always available throughout the testing in P1 and P2. In P3, there was sufficient water during regular operation, but the CWT was drained on three occasions due to leakages from the toilet cistern (days 0–7) and again due to an operational error (days 32–34: BAMBi permeate not pumped into CWT).

The flows through the membrane were measured at the beginning and at the end of the field tests and did not change over the testing period (Table 2). The high fill volumes in the CWT were linked to high water flows in P2 and P3 (respectively 105 and 122 L/h). The flow rate in P1 was considerably lower (3 L/h). A difference remained even when corrected for differences in membrane surface and water pressure in the BAMBi. Clearly, the flow rate was significantly higher than the water usage for P2 and P3, and a substantial part (∼98%) of the recycled water was directly recirculated back to the BAMBi. For improved energy efficiency, a float switch could be implemented in the CWT, stopping the flow from the BAMBi to the CWT when the CWT is full.

**Table 2:** Flow rate and flux (average of values measured at the beginning and end of the field tests) of water pumped into the clean water tank (CWT).

	P1 Hand Washing Station Zurich	P2 Hand Washing Station Durban	P3 Toilet System Durban
Flow rate [L/h]	3	105	122
Flux [L/m^2^·h]	1 (A_membrane_ = 3 m^2^)	6.6 (A_membrane_ = 16 m^2^)	7.7 (A_membrane_ = 16 m^2^)
Specific flux [L/m^2^·h·m]	2 (h_water_ = 0.5 m)	8.2 (h_water_ = 0.8 m)	9.5 (h_water_ = 0.8 m)

Regular water refills were necessary to balance losses due to evaporation, water removed on people’s hands and accidental losses due to leakages. For P3, a substantial amount of water was also lost to the urine diversion of the UDT (∼0.1 L/flush, unpublished laboratory results) and by withdrawal with the solids (∼0.5 L/flush, calculated from water refills and usage). The weekly refill volumes, corrected for accidental losses, were 3.5 L/week for P1, 10 L/week for P2 and 50 L/week for P3.

**Fig. 4:**
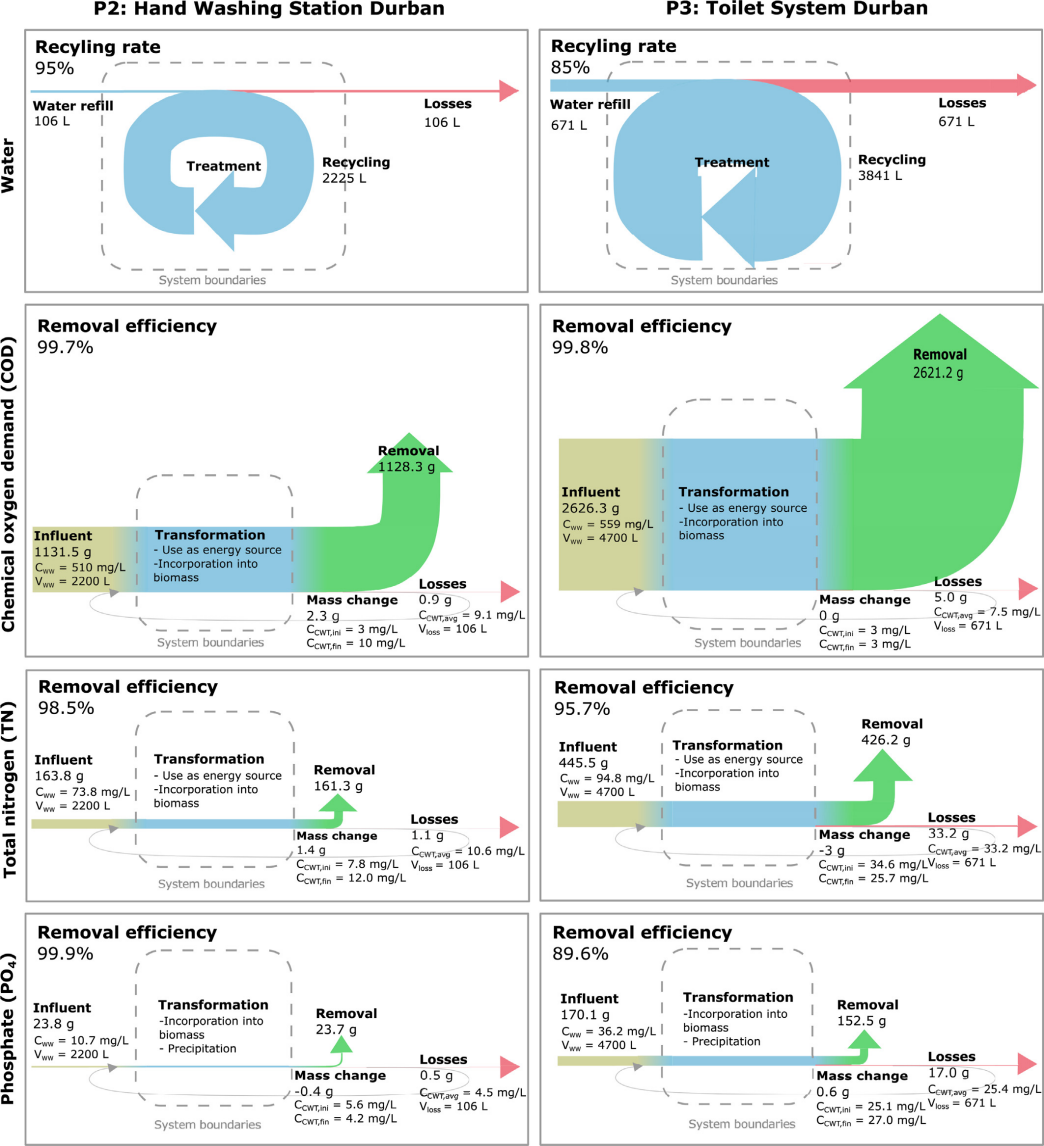
Sankey diagrams with water recycling rate, volume/mass flows and removal efficiencies for chemical oxygen demand (COD), total nitrogen (TN) and phosphate (PO_4_) for the overall testing period between the start and the end of the field trials in P2 and P3. For P1, the water recycling rate was 98% (Supplementary Information Fig. S4). Removal efficiencies could not be calculated for P1, as the influent loads were not available. C_WW_: concentration in the wastewater; V_WW_: total volume of wastewater; C_CWT(ini/fin/avg)_: initial, final and average concentration in the clean water tank (CWT); V_loss_: total volume of water lost from the system (removed on people’s hands, lost to other treatment chains, evaporation).

Comparing the total water usage and loss as described in Equation (1), the water recycling rates were 98% for P1 (Supplementary Information Fig. S4), 95% for P2 and 85% for P3 (Fig. 4).

#### 3.1.2 Chemical composition and nutrient removal

**Fig. 5:**
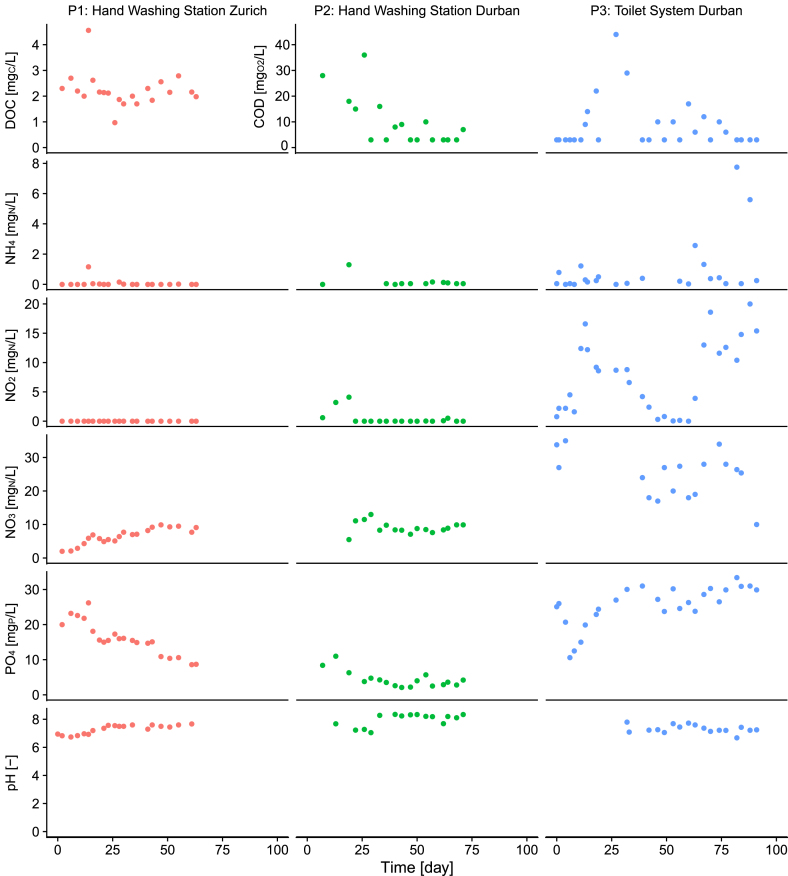
Chemical composition of the recycled water in the clean water tank: dissolved organic carbon (DOC), chemical oxygen demand (COD), ammonium (NH_4_), nitrite (NO_2_), nitrate (NO_3_), phosphate (PO_4_) and pH. On the y-axis, 0 corresponds to measurements below the detection limits.

Fig. 5 shows the concentrations of DOC (P1), COD (P2 and P3), nitrogen compounds (ammonium, nitrite and nitrate), phosphate and the pH in the CWT.

In P1, the average DOC concentration in the CWT was 2.2 ± 0.7 mg_C_/L. The maximum value of 4.6 mg_C_/L on day 14 was due to the loss of 20 L of water (around a third of the total water volume) caused by an act of vandalism. However, the system recovered quickly and DOC concentrations had already dropped to the long-term average by the next day. The average ammonium concentration was 0.07 ± 0.25 mg_N_/L, with a maximum value of 1.16 mg_N_/L after the water loss. Nitrite concentrations were consistently below the detection limit (2 mg_N_/L), while nitrate concentrations initially increased in P1 and stabilized around 9 mg_N_/L. Phosphate concentrations decreased from 20 to 8.7 mg_P_/L over the testing period. The pH was stable (7.3 ± 0.3).

In P2, the average COD concentration was 9.1 ± 10.0 mg_O2_/L. Ammonium was on average 0.16 ± 0.35 mg_N_/L, with an ammonium spike on day 19 (1.3 mg_N_/L) caused by the breakdown of the electrolysis recirculation pump. Nitrite stabilized around the detection limit (0.002 mg_N_/L) after an initial increase up to 4.1 mg_N_/L, while nitrate concentrations were stable around 9.1 ± 1.8 mg_N_/L. Phosphate concentrations decreased from 8.4 to 4.2 mg_P_/L. The pH was stable (8.0 ± 0.5).

In P3, the average COD concentration was 7.5 ± 9.9 mg_O2_/L. The ammonium concentration was on average 0.93 ± 1.89 mg_N_/L, with generally increased concentrations after day 63 (maximum on day 82: 7.75 mg_N_/L). These increased concentrations coincided with an observed reduction in the aeration rate after a power outage on day 63. Nitrite initially increased up to 16.6 mg_N_/L (day 13), after which the concentrations gradually decreased to the detection limit (0.002 mg_N_/L, after day 50). However, a second strong increase was observed starting on day 63 (power outage), after which nitrite concentrations remained high (up to 20 mg_N_/L). Nitrate was stable with an average concentration of 24.6 ± 6.8 mg_N_/L. Only the last measurement indicates a decrease in the nitrate concentration. Phosphate concentrations stabilized at around 30 mg_P_/L. The pH was stable (7.3 ± 0.3).

The mass flows and removal efficiencies of COD, total nitrogen and phosphate in P2 and P3 were calculated for the entire duration of the field trials (Fig. 4). Fig. 4 also presents the average concentrations in the wastewater and CWT. For P2, COD removal was 99.7%, total nitrogen removal 98.5% and phosphate removal 99.9%. P3 had 99.8% COD removal, 95.7% total nitrogen removal and 89.6% phosphate removal. Removal from the water included the use of a compound as energy source, incorporation into the biomass and precipitation in the system. Phosphorus in particular was presumably substantially removed through inorganic precipitation. This indicates that the removal of precipitates from the system may be necessary in longer-term operation in order to ensure a stable system operation. Mass flows and removal efficiencies could not be calculated for P1, as the influent load was not available.

#### 3.1.3 Hygiene

**Fig. 6:**
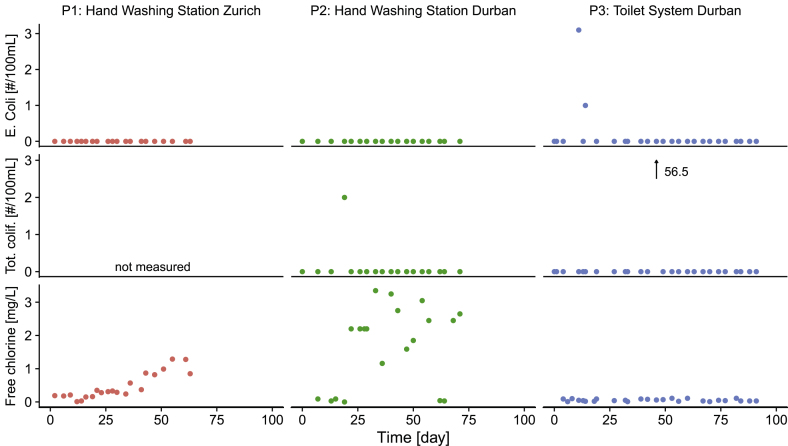
Hygienic quality of the recycled water in the clean water tank: *E. coli*, total coliforms, free chlorine concentration in the clean water tank. *E. coli* as colony-forming units (CFU) for P1. *E. coli* and total coliforms as most probable number (MPN) for P2 and P3. For the bacterial indicators, 0 on the y-axis corresponds to non-detection.

Fig. 6 shows the hygienic quality in the CWT in terms of two pathogen indicators (*E. coli* and total coliforms) and free chlorine concentrations.

In P1, no *E. coli* were detected during the testing (total coliforms not measured). Residual free chlorine concentrations were slightly below 0.2 mg/L (minimum concentration at tap proposed by WHO, 2017) during the first three weeks of operation in P1. There were two occasions with no residual chlorine (day 12: breakdown of the electrolysis recirculation pump, day 14: water loss from vandalism). After an increase in the electrolysis operating time (day 21: 60/90 s on/s off to 60/60 s on/s off), free chlorine concentrations were consistently above 0.2 mg/L. The average free chlorine concentration was 0.5 mg/L (hydraulic retention time (HRT) in the CWT = 400 min) when electrolysis was working.

In P2, no *E. coli* were detected during the testing, but 2 MPN (most probable number)/100 mL total coliforms were measured on day 19. Free chlorine concentrations were below 0.2 mg/L during the first three weeks of operation and on days 63–65 (breakdown of the electrolysis recirculation pump). After the replacement with a pump generating a higher flux (0.5 L/min to 3 L/min), the average free chlorine concentration was 2.2 mg/L (HRT = 23 min) when electrolysis was working.

In P3, *E. coli* concentrations of 3.1 MPN (most probable number)/100 mL and 1 MPN/100 mL were measured on days 11 and 14 respectively. On day 46, a concentration of 56.5 MPN/100 mL total coliforms was measured. Free chlorine concentrations were below 0.2 mg/L throughout the testing. The electrolysis pump broke down on days 36 and 56 and was only replaced 7 and 3 days after the breakdowns respectively. The introduction of a pump with a higher flux on day 43 did not increase the chlorine concentration. The average free chlorine concentration when electrolysis was working was only 0.05 mg/L (HRT = 20 min). We believe the increased total coliforms count on day 46 may be due to contamination during the sampling, as there was residual chlorine in the water (0.1 mg/L free, 0.4 mg/L total) on that day, though microbial regrowth in the system after the prolonged period without electrolysis post-treatment cannot be excluded.

Chlorine was produced from chloride dissolved in the water. In P1, chloride concentrations were stable at around 120 mg/L throughout the testing. In P2 and P3, chloride concentrations initially decreased and required balancing through the addition of NaCl (on average, 3.4 g NaCl/day in P2 and 2.4 g NaCl/day in P3), resulting in average concentrations of 380 and 180 mg/L of chloride in the CWT.

#### 3.1.4 Appearance

**Fig. 7:**
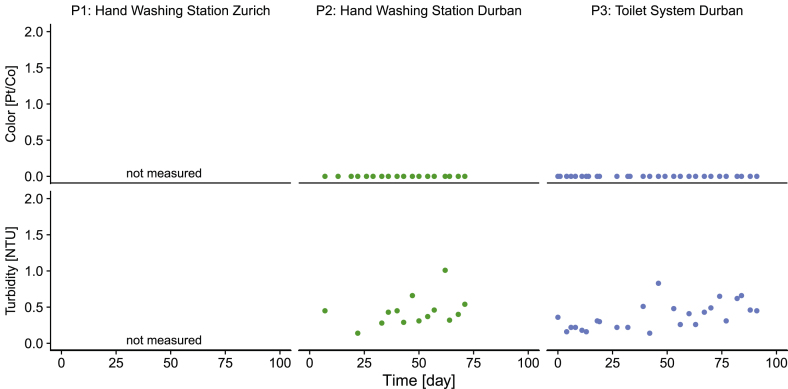
Appearance of the recycled water in the clean water tank: color and turbidity. (For interpretation of the references to color in this figure legend, the reader is referred to the Web version of this article.)

The water was evaluated in terms of color, turbidity and TSS and always appeared clear to the human eye in all field tests. Color measurement revealed 0 Pt/Co (Platinum–Cobalt color scale) throughout the testing of P2 and P3 (Fig. 7). Turbidity was on average 0.44 ± 0.21 NTU in P2 and 0.37 ± 0.18 NTU in P3. As only low TSS were expected after the ultrafiltration membrane, TSS were measured in only two spot samples, revealing a TSS of 0.4 mg/L in P2 and 1.7 mg/L TSS in P3. A visual inspection of the residue indicated that TSS were due to the passage of GAC particles into the CWT.

#### 3.1.5 Energy requirements

The average energy requirements were 0.5 kWh/day for P1 and 1.2 kWh/day for P2 and P3. Taking into account the water production presented in Table 2, this corresponded to around 6.9 kWh/m^3^ in P1 (low water flux), 0.5 kWh/m^3^ in P2 and 0.4 kWh/m^3^ in P3 (both high water fluxes). As reference, the energy requirements for the overall centralized water system for transport and drinking and wastewater treatment is on the order of 1–10 kWh/m^3^ (Olsson, 2015).

### 3.2 Comparison with specifications for wastewater reuse

Table 3 compares the composition of the recycled water with quality requirements for toilet flushing from 18 selected wastewater reuse specifications (details in the Supplementary Information Table S5). Most requirements related to wastewater reuse for toilet flushing were met in all three field trials. Only requirements on chlorine (P1, P2 and P3) and ammonium (P3) were mostly not met. Total phosphorus requirements were partially met (P1, P2 and P3).

**Table 3:** Comparison of monitoring results with quality requirements for toilet flushing from 18 selected wastewater reuse specifications (details in Table S5). The type of requirement (max/min concentration, max/min average, or min removal) is specified in brackets. The column “quality requirements met” describes the percentage of requirements that were met (compared to the number of specifications that give requirements for the parameter) and is categorized into all (100%), mostly (≥90%), partially (≥50%), mostly not (<50%) or none (0%) of the specifications were met.

Parameter	Range of requirements	Quality requirements met		
		P1 Hand Washing Station Zurich	P2 Hand Washing Station Durban	P3 Toilet System Durban
**Chemical composition**				
COD (mg_O2_/L)	50-100 (max), 50 (max avg)	n/a	all	all
TOC^a^ (mg_C_/L)	5 (max), 3 (max avg)	all	n/a	n/a
Total nitrogen (mg_N_/L)	1.6–7.8 (max), 70% (min removal)	all	all	partially
Ammonium (mg_N_/L)	6 (max), 4 (max avg)	all	all	mostly not
Total phosphorus (mg_P_/L)^b^	0.2 (max), 80% (min removal)	partially	partially	partially
pH (−)	6–9.5	all	all	all
**Hygiene**				
Residual chlorine (mg/L)	0.2–5 (min), 0.5 (min avg)	none	mostly not	none
E. coli (#/100 mL)	<1–200 (max), <1–20 (max avg)	all	all	all
Total coliforms (#/100 mL)	0.3–240 (max), 2.2–23 (max avg)	n/a	all	all^c^
**Appearance**				
Color (Pt/Co)	15-30 (max)	n/a	all	all
Turbidity (NTU)	0.5–10 (max), 0.2–5 (max avg)	n/a	mostly	mostly
TSS (mg/L)	5-1500 (max)	n/a	all	all

a TOC ≈ DOC.

b Measured phosphate.

c Excluding one sample that was presumably contaminated during sampling or analysis.

### 3.3 Performance compared to other on-site wastewater reuse systems

Table 4 presents the effluent characteristics and nutrient removal efficiencies in several on-site wastewater reuse systems. With the exception of Rogers et al. (2018), all results presented are from field trials. From the systems investigated, only Cid et al. (2018) and Rogers et al. (2018) achieved true recycling (blackwater reused for toilet flushing). Ratanatamskul et al. (2014) and Sahondo et al. (2020) specify water reuse for toilet flushing as a potential application of the system and Abegglen et al. (2008) reused 35% of the water for toilet flushing and irrigation. Fountoulakis et al. (2016) and Santasmasas et al. (2013) reused treated greywater for toilet flushing (downcycling).

**Table 4:** Characteristics of the treated water and removal efficiencies in six on-site wastewater reuse systems compared to the results presented in this study. Rogers et al. (2018) and Sahondo et al. (2020) tested different configurations (recycling in laboratory testing, sewer discharge in field testing) of the same blackwater treatment system.

Authors/Prototype	This study: P1	This study: P2	This study: P3	Cid et al. (2018)	Rogers et al. (2018)	Abegglen et al. (2008)	Fountoulakis et al. (2016)	Santasmasas et al. (2013)	Sahondo et al. (2020)	Ratanatamskul et al. (2014)
Recycling or downcycling	Recycling	Recycling	Recycling	Recycling	Recycling	Downcycling >65%	Downcycling	Downcycling	Once-through	Once-through
Water treated	Greywater	Greywater	Blackwater^a^	Blackwater	Blackwater^a^	Blackwater	Greywater	Greywater	Blackwater^a^	Blackwater
Reuse purpose	Hand washing	Hand washing	Flushing	Flushing	Flushing	Flushing + irrigation	Flushing	Flushing	Flushing	Flushing + irrigation
Energy	6.9 Wh/L	0.5 Wh/L	0.4 Wh/L	35 Wh/L	5.6 Wh/L (disinfection only)	n.a.	4.2 Wh/L	2.9 Wh/L	15.2 Wh/L	n.a.
Testing context	Public park, Switzerland	Public street, South Africa	Single household, South Africa	Elementary school, China	Laboratory-testing	Single household, Switzerland	Single household, Greece	Office building, Spain	Ablution block, South Africa	University building, Thailand
Duration (days)	63	74	94	30	40-120 UDE^b^	100	400	250	92	105
**Chemical composition of the treated water: mean (min-max)**										
COD (mg_O2_/L)	5.5 (2.5–11.5)^d^	9.1 (<3–36) 99.7% removal	7.5 (<3–44) 99.8% removal	170 (100–300) 70% removal^c^	945 (579–983)	38.1 93% removal	59 (34–157) 87% removal	29 (5–74) 90% removal	61 ± 49 85% removal	<10 >90% removal
Total nitrogen (mg_N_/L)		9.1 (7.4–12.1) 98.5% removal	33.2 (19–44) 95.7% removal				20 (8–47) 40% removal	22 (14–30) 4% removal	102 ± 20 45% removal	
NH_4_ (mg_N_/L)	0.07 (<0.015–1.2)	0.16 (<0.02–1.3)	0.93 (<0.02–7.8)	150 (60–375) 70% removal^c^		1.04 99% removal				1 >90% removal
NO_3_ (mg_N_/L)	6.5 (2–9.9)	5.5 (10–13)	24.6 (10–35)			45	0.2			25
Total phosphorus (mg_P_/L)	16 (8.6–26.2)^e^	4.4 (2.1–11.0) 99.9% removal^e^	25.4 (10.6–33.4) 89.6% removal^e^			17 29% removal	0.4 (0.1–1.6) 69% removal	3 (2–8)	14 ± 12 20% removal	0.3
pH (−)	7.3 (6.7–7.7)	8.0 (7.1–8.4)	7.3 (6.7–7.8)				7.9 (7.0–8.6)	7.9 (7.5–8.3)	7.0 ± 0.6	7.5–8.3
EC (us/cm)		2490 (2060–2870)	1480 (720–1900)		15,000 (14,000-16,400)	38.1 93% removal	800 (600–1900)	1240 (930–1630)		
**Pathogen indicators in the treated water: mean (min-max)**										
E. coli (MPN/100 mL)	<1	<1	<1 (<1–3.1)	<1			<1	<5 (<5–100, CFU/100 mL)	<1	<1
Tot. Coliforms (MPN/100 mL)		<1 (<1–2)	<1 (<1–56.5)	9 (max. after 4 h disinf.)			1.3 (<1–18)		<1	
**Appearance of the treated water: mean (min-max)**										
Color (Pt/Co)		0	0		86 (25–150)				90 max	
Turbidity (NTU)		0.4 (0.1–1)	0.4 (0.1–0.8)		155 (106–192)		5.0 (1.5–9.9)		3 ± 1 97% removal	0–0.1
TSS (mg/L)		0.4	1.7		138 (52–216)		8 (4–12)	1.3 (<1–5)	23 ± 13 65% removal	

a Blackwater separated from the major part of urine and solids (this study) or solids (Sahondo et al., 2020; Rogers et al., 2018).

b UDE: user-day equivalent.

c Concentrations: estimated from Fig. 7 in Cid et al. (2018). Removal: comparison between input and output concentrations; not all data available to calculate overall removal for the complete field trial duration.

d Assumed COD/DOC = 2.5 g_O2_/g_C_.

e Phosphate.

## 5 Discussion

### 5.1 Appealing water free of bacterial pathogen indicators, but sensitive to failure of electrolysis

All prototypes tested in this study were capable of producing sufficient quantities of appealing water throughout the field trials. Usage of all systems was high through the testing period despite alternatives being available in the immediate surroundings and no concerns were raised by users regarding the quality of the recycled water. It hence appears that users deemed the water suitable for hand washing and toilet flushing. The laboratory analyses confirm that the Water Wall systems effectively removed chemical contaminants, visual impurities and bacterial pathogen indicators from the used water in real-life hand washing and toilet flushing applications.

The field trials also allowed for the identification of weaknesses and components prone to failure. Electrolysis for disinfection and to produce a chlorine residual was identified as the most vulnerable process in the treatment chain. Chlorine concentrations were affected by the following three mechanisms.

First, the failure of components, specifically the electrolysis recirculation pump, resulted in the depletion of the residual chlorine in the CWT. When assembling the prototypes, one of the objectives had been to reduce the cost of individual components. However, these issues highlight the necessary tradeoff between increasing the capital costs of a system with more robust components and increasing the maintenance costs (or decreasing the system reliability) due to more frequent replacement of system parts.

Second, increased concentrations of ammonia and nitrite in the influent to the electrolysis unit impaired the efficacy of the disinfection process. Over the last four weeks of the field trial, reduced nitrification in P3 was observed, caused by a reduced aeration rate due to a partial blockage of the aeration tubes after a power outage. Reduced nitrification resulted in increased nitrite and ammonium concentrations. While nitrite and ammonium are not per se a constraint for reuse as flush water (as risk of ingestion is minimal), both compounds affect the efficacy of the electrolysis post-treatment as they react with the residual free chlorine to form nitrate (from nitrite) and chloramines (from ammonium) (Yang and Cheng, 2007). Many developing countries suffer from severe electricity deficits (Seetharam et al., 2013). Our field trials revealed that power cuts might have a long-term impact on the water quality. A backup power supply system (for instance with solar panels and batteries) would increase system robustness, but is associated with increased capital costs. Again, we believe there is need for a detailed tradeoff analysis for the specific setting. For instance, low maintenance may be more critical in a household than in a public setting (possibly with several Water Wall systems), where maintenance personnel can be available on-site more frequently.

Third, there was a need for chloride supplementation in P2 and P3 by adding NaCl to ensure stable chlorine production from chloride. This had not been expected as chloride additions (from soap, people’s hands and urine contamination) had balanced the chlorine losses due to volatilization in previous testing. While the supplemental chloride can be added through the soap in the case of hand washing water (P2), the need for chloride supplementation is a disadvantage for systems recycling flush water (P3) that would otherwise not have required any additives. There are two approaches for ensuring hygiene with these described potential failure modes - process simplification or increased monitoring capability: (i) Electrolysis could be replaced by the addition of slow-dissolving chlorine tabs (rather than the addition of NaCl for on-site chlorine production), making chlorination independent of the availability of power. Previous research indicates that electrolysis treatment does not yield lower cell counts than direct chlorination (Ziemba et al., 2019), implying that direct chlorine addition disinfects the water as effectively as electrolysis treatment. There would, however, be the drawback of reliance on supply chains and maintenance, though this is true of any spare parts or consumables. (ii) More advanced monitoring of unit processes and residual chlorine to provide real-time information on water safety would prevent the use of potentially compromised water. Online monitoring cannot prevent all forms of process failures, but it can shut off the system in case a problem is detected.

While the Water Wall uses high-tech components, the system was designed with robust operation and low maintenance requirements in mind. The aerobic bioreactor was operated without the need for sludge wastage. The ultrafiltration membranes produced a stable flux without the need for shear, cleaning, or backwashing. The GAC filter did not require replacement. The electrolysis unit was operated with simple timer control and polarity reversal to prevent scaling. Out of these four barriers for chemical and microbial contaminants, only the electrolysis was prone to failure.

### 5.2 True water recycling possible due to source separation of urine and feces

The reuse applications of recycled wastewater depend on the water quality that can be achieved. True recycling for toilet flushing is not yet well established and most systems in Table 2 downcycled the water, likely in part because of the ‘yuck’ factor (Smith et al., 2018) or diluted the treated water with freshwater, which can allow repeated reuse whilst minimizing nutrient build-up in the system.

In Cid et al. (2018) and Rogers et al. (2018), the blackwater recycling systems experienced high nutrient concentrations in the treated water, with COD concentrations over 900 mg/L and ammonia concentrations over 100 mg/L. Non-biological treatment processes generally struggle to meet nutrient removal targets (e.g., 80% phosphorus removal in ISO 30500) without the use of chemical or biological additives (Trotochaud et al., 2019).

In contrast, the prototypes tested as part of the current study all produced recycled water with low and stable organic carbon and nutrient concentrations. Avoiding the accumulation of nitrogen in the recycling of toilet flush water (P3) was due to nitrification and denitrification in the BAMBi and due to the fact that the major part of the urine was separated at the source. Source separation of urine limited the amount of nitrogen entering the system and generated COD/N ratios suitable for denitrification. If all the urine and fecal matter had entered the flush water, this would have resulted in a COD/N ratio in the order of 3:1. However, a minimum COD/N ratio of 7.6:1 has been reported as necessary for full denitrification (Sobieszuk and Szewczyk, 2006). Similarly, phosphorus concentrations would have increased had major parts of the feces not been diverted. The separation of urine and feces from the water also led to considerably reduced energy requirements compared to Cid et al. (2018), Sahondo et al. (2020) and even compared to centralized systems (Olsson, 2015). Besides the efficient recovery of the water, the implementation of source separation also enables the recovery and reuse of nutrients from feces and urine (Larsen et al., 2009; Zeeman and Kujawa-Roeleveld, 2011). We believe that separating water, urine and feces is a vital requirement for flush water recycling, allowing for an optimized recovery of resources from each stream.

### 5.3 Wastewater reuse specifications should be risk-based

The required level of treatment and treatment targets should be linked to the intended reuse application, as health risks associated with wastewater reuse depend on likely contact pathways. Today, many specifications on non-potable wastewater reuse do not differentiate between specific reuse applications. The category “unrestricted urban reuse” used by US EPA (2012), for instance, may comprise landscape irrigation as well as toilet flushing. Only the ISO standard 30,500 explicitly mentions hand washing as a potential application of recycled water (ISO, 2018), but does not provide specific targets and instead refers to the World Health Organization guidelines for drinking water (WHO, 2017).

Because true recycling for high quality applications is not yet well established, many wastewater reuse specifications target applications where the water is downcycled and ultimately released into the environment (e.g., irrigation). Many quality requirements from such specifications are, however, unsuitable for true recycling applications where there is no discharge. For instance, the Water Wall systems did not meet the requirements on (maximum) chloride concentrations (Table S4; presumably to protect receiving water bodies), although elevated chloride concentrations are necessary for chlorine production in this technology. From our perspective, specifications should clearly differentiate between downcycling and recycling wastewater reuse systems, as the latter do not need to account for ecosystem impacts.

Especially for applications involving close personal contact with the water, like the Water Wall system, the effective protection of human health is essential. We see two limitations in the evaluation of hygiene presented in this paper.

1. Today, many specifications assess the microbial quality of the treated water in terms of bacterial fecal contamination and require monitoring of indicators like *E. coli* or total coliforms (Table S4). The Water Wall met the criteria of safety for fecal bacterial contamination, but this approach may be insufficient, due to (i) limited fecal contamination in the source water in P1 and P2 (hand washing water), and (ii) microbial risk that may be linked to regrowth during storage rather than incomplete retention. In membrane bioreactors, most bacteria are retained by the membrane (>6-log for ultrafiltration, Sharvelle et al., 2017), but there can be regrowth of bacteria in the CWT in the absence of residual chlorine (Nguyen et al., 2017). We thus have doubts regarding the appropriateness of fecal bacteria as the main indicator of hygiene in hand washing systems and more generally, in systems that rely on ultrafiltration processes. For such systems, we need to identify appropriate indicators for hand washing water and for regrowth in the stored water. In systems with final disinfection, like the Water Wall, it may also be appropriate to measure disinfectant concentrations (e.g., residual chlorine) if the required doses for the inactivation of the system-relevant indicator organisms have been established.
2. Despite their relevance for public health, many wastewater reuse specifications do not define virus-related quality requirements. For instance, only 2 out of 10 guidelines for unrestricted urban reuse in the United States include virus removal rates (prescribed by the treatment requirements, Tables 4–7, US EPA, 2012). The ultrafiltration membrane used in the Water Wall with a molecular weight cutoff of 150 kDA cannot guarantee sufficient virus retention (log-removal < 0.5 for the bacteriophage MS2, unpublished results). Their inactivation is thus dependent on the final disinfection with chlorine, which emerged as the most vulnerable treatment step during the field tests. Monitoring viruses is thus highly relevant for membrane systems like the Water Wall. It is possible to define virus-related indicators and many studies have proposed the use of native coliphages (bacteriophages that infect *E. coli*) as indicators of enteric viruses (Zhang and Farahbakhsh, 2007). However, there is limited practical experience with virus monitoring in real-life implementations of on-site wastewater reuse systems. Another strategy is to evaluate virus removal by spiking known amounts of virus surrogates into the system, which is required by some wastewater reuse specifications (most commonly bacteriophage MS2, see Table S4). Future research should focus on assessing to what degree viral indicators (versus virus challenge studies) are useful in assessing safety of water reuse for non-potable purposes (e.g., hand washing).

Overall, we believe there is a need for a paradigm shift from “one-fits-all” wastewater reuse specifications that do not differentiate between types of water sources and end uses, to more flexible approaches enabling a pragmatic development of water recycling systems that ensure the reliable delivery of safe water for a specific context. The WERF report by Sharvelle et al. (2017) has made an important step towards the implementation of risk-based (and thus context-based) performance targets by publishing a framework for the development of public health guidance for decentralized water reuse systems. Such a framework for water quality targets and technology choices needs to be coupled with an organization, maintenance and monitoring approach that ensures system performance. There is a need for adequate online measurements that are reliable, effective and correlated with system performance, to protect public health. Future research on on-site wastewater reuse needs to focus on the identification and evaluation of such monitoring devices, thus allowing for direct intervention in case of treatment failure.

## 6 Conclusions

‐ The field trials of Water Wall prototypes in Switzerland and South Africa show that the system effectively removes chemical contaminants, visual impurities and pathogen indicators from the used water. Low and stable DOC, COD, nitrogen and phosphorus concentrations in the treated water allowed for true recycling of the water, which distinguishes the Water Wall systems from comparable wastewater reuse systems that mostly downcycle the water. When treating flush water, removal of nutrients to ensure true recycling water quality was possible due to reduced influent contaminant concentrations, as the urine and the major part of the particles had been separated before the treatment.
‐ Throughout the testing, the prototypes were capable of providing sufficient quantities of flush and hand washing water, where systems were evaluated at different scales, in varied geographical contexts and in public and private applications. Usage of all systems was frequent despite alternatives being available in the immediate surroundings.
‐‐ Overall, our results from real-life applications show that the technology is ready to move forward towards a larger-scale implementation of prototypes under the lead of an entity that would be specialized in the detailed engineering, construction and marketing of such systems.
‐ To ensure effective protection of human health, we advocate for the implementation of risk-based (and thus end-use dependent) system performance targets for future evaluation of such systems.

## Declaration of competing interest

The authors declare that they have no known competing financial interests or personal relationships that could have appeared to influence the work reported in this paper.
