# Supplementary material for: Practical implementation of true on-site water recycling systems for hand washing and toilet flushing
Source: Water Res X. 2020 Apr 8;7:100051. doi: 10.1016/j.wroa.2020.100051 (PMC7242789; doi:10.1016/j.wroa.2020.100051)
Supplement: Multimedia component 1 [file mmc1.pdf]

Practical implementation of true on-site water recycling systems for hand washing and toilet flushing

Supplementary Information

Eva Reynaert[a], Esther E. Greenwood[a], Bonginkosi Ndwandwe[c], Michel E. Riechmann[a],

Rebecca C. Sindall[c], Kai M. Udert[a, b] and Eberhard Morgenroth[a, b, *]

[a] Eawag, Swiss Federal Institute of Aquatic Science and Technology, 8600 Dübendorf, Switzerland

[b] ETH Zürich, Institute of Environmental Engineering, 8093 Zürich, Switzerland

[c] University of KwaZulu Natal, Pollution Research Group, 4041 Durban, South Africa

*Corresponding Author: Eberhard.Morgenroth@eawag.ch

Reynaert, E., Greenwood, E. E., Ndwandwe, B., Riechmann, M. E., Sindall, R. C., Udert, K. M. & Morgenroth, E. 2020. Practical implementation of true on-site water recycling systems for hand washing and toilet flushing. Water Research X, https://doi.org/10.1016/j.wroa.2020.100051.

Supplementary information 1: Nutrient-supplemented soap

Table S1. Composition of the soap used in the field test. The strategy to add nutrients is described in Ziemba et al. (2018) with only 30% of the suggested nutrients added (based on laboratory testing). Prior to use, the soap was diluted 10x in a foaming soap dispenser.

Nutrient	Compound	Recipe g/L
C	SDS	140
C	Glycerol	50
C	Lactic acid	0.72
	NaCl	10
N	NH_4NO_3	12.3
N	$NaNO_3$	13.1
P	HNa2O4P·$2H_2O$	9.3
S	Na_2SO_4	
K	KCl	1.0
Mg	$Cl_2Mg \cdot 6H_2O$	2.3
Ca	$CaCl_2$	1.5
Fe	$Cl_2Fe \cdot 4H_2O$	1.0
Mn	$MnSO_4 \cdot H_2O$	0.003
Cu	$CuCl_2 \cdot 2H_2O$	0.005
Zn	$ZnCl_2$	0.009
Mo	$MoCl_5$	0.0004
Co	$Cl_2Co \cdot 6H_2O$	0.0006

Supplementary information 2: Photographs

P1: Hand Washing Station Zurich

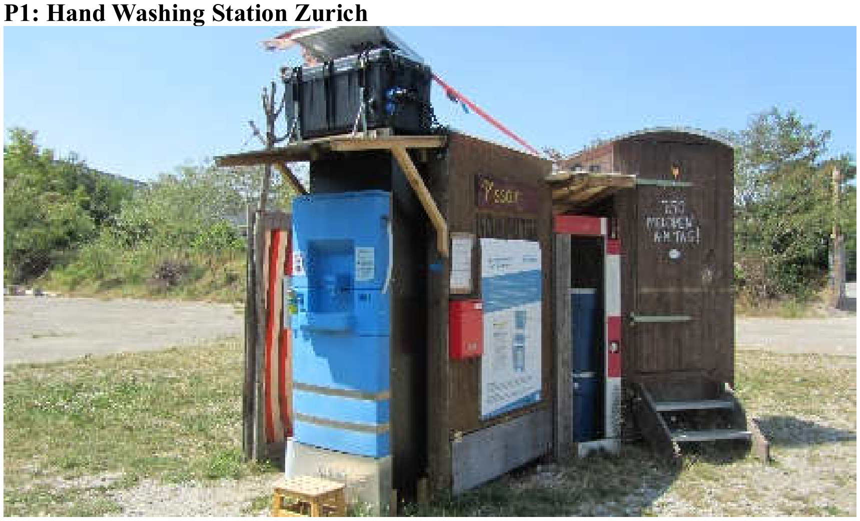

P2: Hand Washing Station Durban

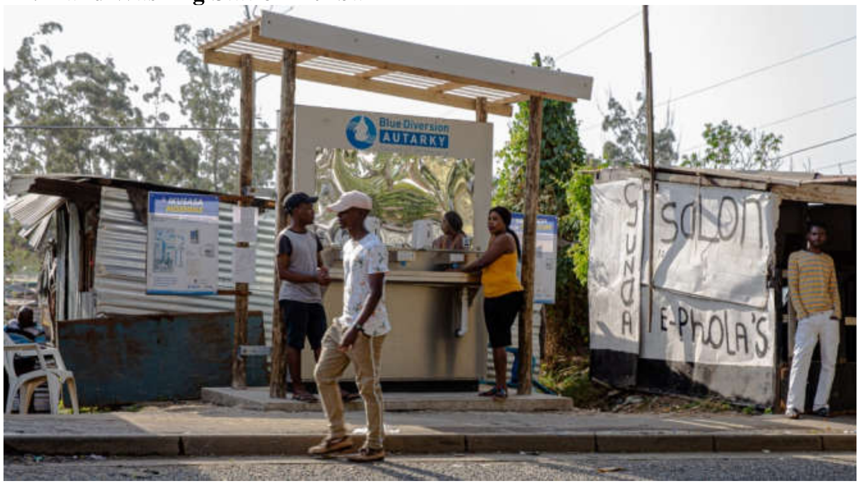

P3: Toilet System Durban

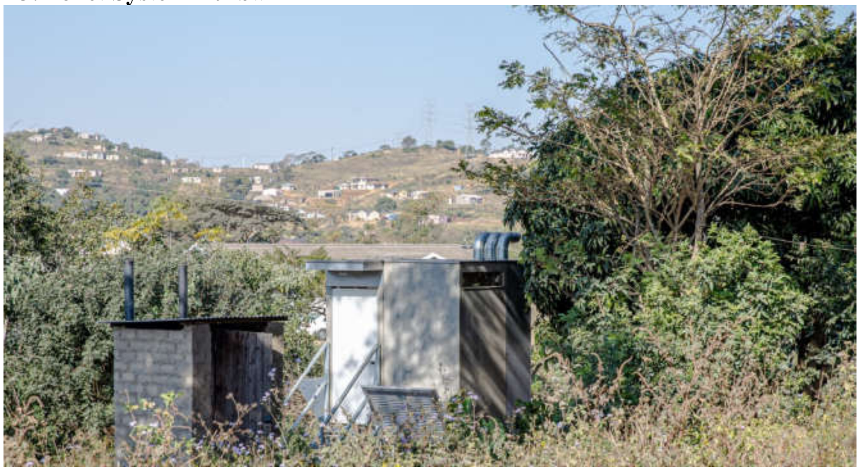

Figure S2. Photographs of the prototypes. P1 (blue) was located next to existing composting toilet facilities. P2 was installed below a shed off the main road. P3 (back) was standing behind an existing dry toilet facility.

Supplementary information 3: Water quality monitoring

Table S3. Overview of spectrophotometric test kits used for water quality monitoring

Parameter	Method
**Chemical water composition**	
Chemical oxygen demand (COD)	P2, P3: Spectrophotometry, COD 0-1500 mg/l, Spectroquant, Merck, Darmstadt, Germany
Ammonium	P1: Spectrophotometry,, LCK 304, Hach, Loveland, USA<br>P2, P3: Spectrophotometry, Ammonium 0.01-2 mg/l, Spectroquant, Merck, Darmstadt, Germany
Nitrite	P2, P3: Spectrophotometry, Nitrite 0.002-1 mg/l, Spectroquant, Merck, Darmstadt, Germany
Nitrate	P2, P3: Spectrophotometry, Nitrate 0.002-1 mg/l, Spectroquant, Merck, Darmstadt, Germany
Total nitrogen	P2, P3: Spectrophotometry, Total nitrogen 0.5-15 mg/l, Spectroquant, Merck, Darmstadt, Germany
Chloride	P2, P3: Spectrophotometry, Chloride 0.1-25 mg/l, Spectroquant, Merck, Darmstadt, Germany
Phosphate	P2, P3: Spectrophotometry, Nitrate 0.002-1 mg/l, Spectroquant, Merck, Darmstadt, Germany

Supplementary information 4: Water recycling rate for P1

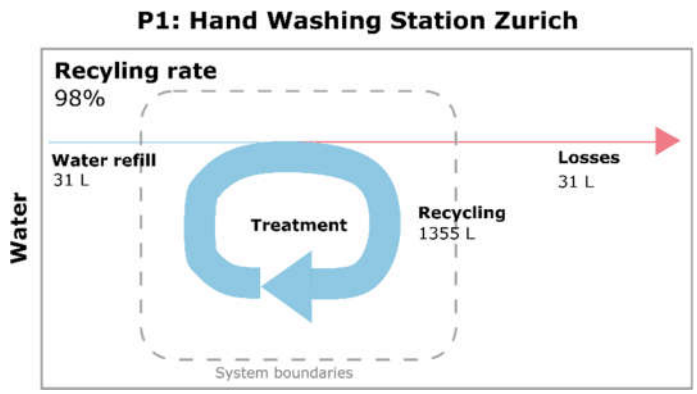

Figure S4. Sankey diagram with water recycling rate and water volume flows for the overall testing period between the start and the end of the field trials in P1.

Supplementary information 5: Specifications on wastewater reuse

Table S5. Overview of several specifications on wastewater reuse for toilet flushing and/or hand washing. HWS: hand washing station (treating greywater). ITS: integrated toilet system (treating toilet flush water after urine and solids separation). ND: not determined. CFU: colony-forming unit. MPN: most probable number. PFU: plate-forming unit. min, max, med, geom: minimum, maximum, median, geometric mean. wk, mon, ann: weekly, monthly, annual.

**Publishing organization**		**ISO**	**State Water Resources Control Board**	**US EPA**											**WHO**	**Environmental Health Directorate**	**Ministry of Health**	**Ministry of the Environment and Rural and Marine Affaires**	**Ministry of the Environment and the Protection of the Territory**	**State Administration of Quality Supervision, Inspection and Quarantine**	**Environmental field testing platform**
**Document**		**ISO 30500: Non-Sewered Sanitation Systems -- Prefabricated Integrated Treatment Units -- General Safety and Performance Requirements for Design and**	**Regulations Related to Recycled Water (Title 22)**	**Guidelines for Water Reuse**											**Guidelines for Drinking-Water Quality**	**Code of Practice for the Reuse of Greywater Guidelines for the Non-potable uses of recycled water**	**Canadian Guidelines for Domestic Reclaimed Water for Use in Toilet and Urinal flushing**	**RD 1620/2007: Spanish Regulations for Water Reuse**	**DM 185/2003: Regulation Containing Technical Rules for the Reuse of Wastewater**	**Municipal Wastewater Recycling: Water Quality Standards for Urban Water Consumption (misc.)**	**Standard for Water to be Recycled as Flushwater from Prototypes on the EFT**
**Year**		2018	2018	2012											2011	2010	2010	2007	2003	2002	2019
**Country/State**		International	USA, California	USA, Arizona	USA, California	USA, Florida	USA, Hawaii	USA, Nevada	USA, New Jersey	USA, North Carolina	USA, Texas	USA, Virginia	USA, Washington	Recommendation of EPA	International	Australia, Western Australia	Canada	Spain	Italy	China	South Africa
Source & reuse purpose	**Water source**	Wastewater	Wastewater	General Wastewater												Greywater	Wastewater	Wastewater	Wastewater	Wastewater	
	**Water source – AUTARKY**	HWS + ITS	HWS+ITS	HWS+ITS	HWS+ITS	HWS+ITS	HWS+ITS	HWS+ITS	HWS+IT	HWS+IT	HWS+ITS	HWS+ITS	HWS+ITS	HWS+ITS		HWS	HWS + ITS	HWS + ITS	HWS + ITS	HWS + ITS	HWS + ITS
	**Reuse purpose**		Toilet flushing	Unrestricted urban reuse	Unrestricted urban reuse	Unrestricted urban reuse	Unrestricted urban reuse	Unrestricted urban reuse	Unrestricted urban reuse	Unrestricted urban reuse	Unrestricted urban reuse	Unrestricted urban reuse	Unrestricted urban reuse	Unrestricted urban reuse	Drinking Water	Toilet flushing, dedicated cold water taps for clothes washing	Toilet flushing	Sanitary appliances	Toilet flushing	Toilet flushing	
	**Reuse purpose - AUTARKY**	HWS + ITS	ITS	ITS (HWS unclear)	ITS (HWS unclear)	ITS (HWS unclear)	ITS (HWS unclear)	ITS (HWS unclear)	ITS (HWS unclear)	ITS (HWS unclear)	ITS (HWS unclear)	ITS (HWS unclear)	ITS (HWS unclear)	ITS (HWS unclear)		ITS + HWS	ITS	ITS (HWS unclear)	ITS (HWS unclear)	ITS	HWS + ITS
Chemical water composition	Chemical oxygen demand COD (mg/L)	<= 50																	<= 100		50 (avg)
	Biological oxygen demand after 5 days BOD5 (mg/L)					<= 20 (ann avg)<br><= 30 (mon avg)<br><= 45 (wk avg)<br><= 60 (max)	<= 30 or 60 depending on design flow	<= 30 (mon avg)		<= 10 (mon avg)<br><= 15 (daily max)	<= 5	<= 10 (mon avg)	<= 30	<= 10			<= 10 (med)<br><= 20 (max)		<= 20	<= 10	5 (avg)<br>20 (max)
	Total organic carbon TOC (mgC/L)									<= 3 (mon avg)<br><= 5 (max)											
	Total nitrogen (mgN/L or removal)	70% removal																	<= 15		
	Ammonium (mgN/ L)								NH3-N+NO3-N < 10 mg/L (max)	<= 4 mon avg)<br><= 6 (daily max)									<= 1.6	<= 7.8	
	Total phosphorus (mgP/L or removal)	80% removal																	<= 0.2		
	Chlorides (mgCl/L)														<= 250				<= 250		
	pH (-)	6-9												6-9		6.5-8.5			6-9.5	6-9	6-9
Hygiene	Residual chlorine (mgCl2/L)					> 1	> 5					> 1	> 1	> 1	>= 0.5<br>>= 0.2 (in pipe)	0.2-2.0	>= 0.5			>= 1 (after 30 min)<br>>= 0.2 (in pipe)	> 0.5 (avg)
	E.coli (MPN or CFU/100mL)	<= 1 CFU<br>>= 6 LRV								<=14 MPN (mon mean)<br><= 25 MPN (max)	<= 20 MPN (30d geom)<br><= 75 MPN (max)	<= 11 MPN (mon geom)<br><= 35 MPN (max)				< 1 (MPN or CFU, med of 6 consecutive samples)	ND (med)<br><= 200 CFU (max)	ND	<= 10 CFU (80% of samples)<br><= 100 CFU (max)		1 (avg)<br>10 (max)
	Fecal coliforms (MPN or CFU/100mL)			ND in least 4 of 7 samples<br><= 23 MPN (max)		75 % of samples ND<br><= 25 MPN (max)	<= 2.2 MPN (7d median)<br><= 23 MPN (not more than 1 sample exceeds this in 30d)<br><= 200 MPN (max)		<= 2.2 MPN (wk med)<br><=14 MPN (max)	<= 14 MPN (mon mean)<br><= 25 MPN (max)	<= 20 MPN (30d geom)<br><= 75 MPN (max)	<= 14 MPN (mon geom)<br><= 49 MPN (max)		ND/100 mL							
	Total coliforms (MPN or CFU/100mL)		<= 2.2 MPN (7 med)<br><= 23 MPN (one sample of 30d)<br><= 240 (max)		<= 2.2 MPN (7-day med)<br><= 23 MPN (not more than one sample exceeds this value in 30 d)<br><= 240 MPN (max)			<= 2.2 MPN (30d geom)<br><= 23 MPN (max)					<= 23 MPN (7d-med)<br><=240 (max)							<= 0.3 (<3 units/L)	
	Coliphage MS2 (PFU/100mL, LRV)	<= 1<br>>=7 LRV	>= 5 LRV (or poliovirus)												<1 (med of 6 consecutive samples)						
Appearance	Colour (Pt/Co)														<= 15					<= 30	
	Turbidity (NTU)		<= 2 (daily avg)<br><= 5 (5% of day)<br><= 10 (max)	<= 2 (daily avg)<br><= 5 (max)	<= 0.2 (avg)<br><= 0.5 (max)	<= 2-2.5			<= 2	<= 10	<= 3	<= 2 (daily avg)<br><= 5 (max)	<= 2 (avg)<br><= 5 (max)	<= 2	<= 1	<= 2 (95% percentile)<br><= 5 (max)	<= 2 (med)<br><= 5 (max)	<= 2		<= 5	5 (avg)<br>10 (max)
	Total suspended solids TSS (mg/L)	<= 10				<= 5	<= 30 or 60 depending on design flow	<= 30 (mon avg)	<= 5	<= 10 (daily max)<br><= 5 (mon avg)			< 30 (max)		<= 15		<= 10 (med)<br><= 20 (max)	<= 10	<= 10	<= 1500	10 (avg)<br>30 (max)

References

Department of Health Western Australia. (2010). *Code of Practice for the Reuse og Greywater in Western Australia.* East Perth, Australia Retrieved from https://ww2.health.wa.gov.au/~/media/Files/Corporate/general%20documents/water/PDF/Code_of_practice_for_the_reuse_of_greywater_in_WA_010_v2_130103.pdf.

Health Canada. (2010). *Canadian Guidelines for Domestic Reclaimed Water for Use in Toilet and Urinal Flushing*. Ottawa, Canada: Canadian Government Retrieved from https://www.canada.ca/content/dam/canada/health-canada/migration/healthy-canadians/publications/healthy-living-vie-saine/water-reclaimed-recyclee-eau/alt/reclaimed-water-eaux-recyclees-eng.pdf.

ISO. (2018). ISO 30500: Non-sewered sanitation systems — Prefabricated integrated treatment units — General safety and performance requirements for design and testing. In. Geneva, Switzerland: International Organization for Standardization (ISO).

Ministry of the Environment and the Protection of the Territory Italy. (2003). *DM 185/2003: Regulation Containing Technical Rules for the Reuse of Wastewater. (Decreto 12 giugno 2003, n. 185: Regolamento recante norme tecniche per il riutilizzo delle acque reflue in attuazione dell'articolo 26, comma 2, del decreto legislativo 11 maggio 1999, n. 152)*. Rome, Italy: Ministry of the Environment and the Protection of the Territory Retrieved from https://www.gazzettaufficiale.it/eli/id/2003/07/23/003G0210/sg.

Ministry of the Presidency Spain. (2007). *RD1629/2007 Spanish Regulations for Water Reuse (Real Decreto 1620/2007, de 7 de diciembre, por el que se establece el régimen jurídico de la reutilización de las aguas depuradas)*. Madrid, Spain Retrieved from https://www.boe.es/eli/es/rd/2007/12/07/1620.

State Administration of Quality Supervision, Inspection and Quarantine China. (2002). *GB/T 18920-2002: Municipal Wastewater Recycling: Water Quality Standards for Urban Water Consumption*. Retrieved from https://webstore.ansi.org/standards/spc/gb189202002.

State Water Resources Control Board California. (2018). *Regulations Related to Recycled Water: Title 22 Code of Regulations*. Sacramento, USA: State Water Resources Control Board Retrieved from https://www.waterboards.ca.gov/drinking_water/certlic/drinkingwater/documents/lawbook/rwregulations.pdf.

US EPA. (2012). *2012 Guidelines for Water Reuse. EPA/600/R-12/618*. Washington, USA: Environmental Protection Agency.

WHO. (2017). *Guidelines for Drinking-water Quality: fourth edition incorporating first addendum* (4th ed.). Geneva, Switzerland: World Health Organization.

Ziemba, C., Larivé, O., Reynaert, E., & Morgenroth, E. (2018). Chemical composition, nutrient-balancing and biological treatment of hand washing greywater. *Water research, 144*, 752-762.
